# Liver-Targeted Nanoparticles Loaded with Cannabidiol Based on Redox Response for Effective Alleviation of Acute Liver Injury

**DOI:** 10.3390/foods13152464

**Published:** 2024-08-04

**Authors:** Xuan Zhang, Xiangzhou Yi, Xia Gao, Yongcheng Li, Xuanri Shen

**Affiliations:** 1 School of Food Science and Engineering, Hainan University, Haikou 570228, China; zhangxuan@hainanu.edu.cn (X.Z.); xiangzhouyi1995@hainanu.edu.cn (X.Y.); gaoxia20202021@163.com (X.G.); lyc2360@sina.com (Y.L.); 2 Key Laboratory of Food Nutrition and Functional Food of Hainan Province, Haikou 570228, China; 3 Hainan Engineering Research Center of Aquatic Resources Efficient Utilization in South China Sea, Hainan University, Haikou 570228, China; 4 College of Food Science and Engineering, Hainan Tropical Ocean University, Sanya 572022, China

**Keywords:** cannabidiol, liver targeting, redox response, acute liver injury

## Abstract

The purpose of this work was to construct liver-targeted nanoparticles based on the redox response to effectively deliver cannabidiol (CBD) for the prevention of acute liver injury (ALI). CBD-loaded nanoparticles (CBD NPs) with a particle size of 126.5 ± 1.56 nm were prepared using the polymer DA-PP-LA obtained by grafting pullulan polysaccharide with deoxycholic acid (DA) and α-lipoic acid (α-LA). CBD NPs showed typical redox-response release behavior. Interestingly, CBD NPs exhibited admirable liver targeting ability, significantly accumulated in the liver, and effectively promoted the internalization of CBD in liver cells, thus effectively reducing the H_2_O_2_-induced oxidative damage of HepG2 cells and avoiding apoptosis. More importantly, CBD NPs effectively prevented CCl_4_-induced ALI by protecting liver function, ameliorating oxidative stress levels, inhibiting the production of inflammatory factors, and protecting the liver from histological damage. This study provides a promising strategy for achieving targeted delivery of CBD NPs in the liver, thereby effectively preventing ALI.

## 1. Introduction

The liver, a crucial metabolic organ in the human body, primarily handles the detoxification of toxic chemicals and drugs [1]. However, the liver is also susceptible to damage from certain toxins, drugs, or chemicals while processing and neutralizing these substances [2]. Of note, recently, chemical liver injury has emerged as a significant threat to human health, with its incidence rate increasing rapidly. Carbon tetrachloride (CCl_4_) is a well-known hepatotoxin frequently employed in experimental models to investigate liver damage [3]. Upon metabolic activation by hepatic cytochrome P450 enzymes, CCl_4_ produces highly reactive trichloromethyl radicals (CCl_3_•) and trichloromethyl peroxy radicals (CCl_3_O_2_•), as well as excess reactive oxygen species (ROS). These reactive substances can damage intracellular macromolecules (e.g., lipids, proteins, and DNA), triggering lipid peroxidation and leading to cell membrane damage, oxidative stress, and subsequent liver injury [4]. Therefore, protecting against CCl_4_-induced ALI involves inhibiting oxidative stress and inflammatory responses. However, currently available treatment methods are limited: (i) Drugs for ALI usually lead to hepatotoxicity and trigger further liver injury [5]. (ii) The only method for more serious ALI is liver transplantation surgery, while also having a greatly limited source of donors [6]. Hence, these points underscore the pressing need to investigate safe and effective new treatments to prevent ALI effectively.

**Scheme 1:**
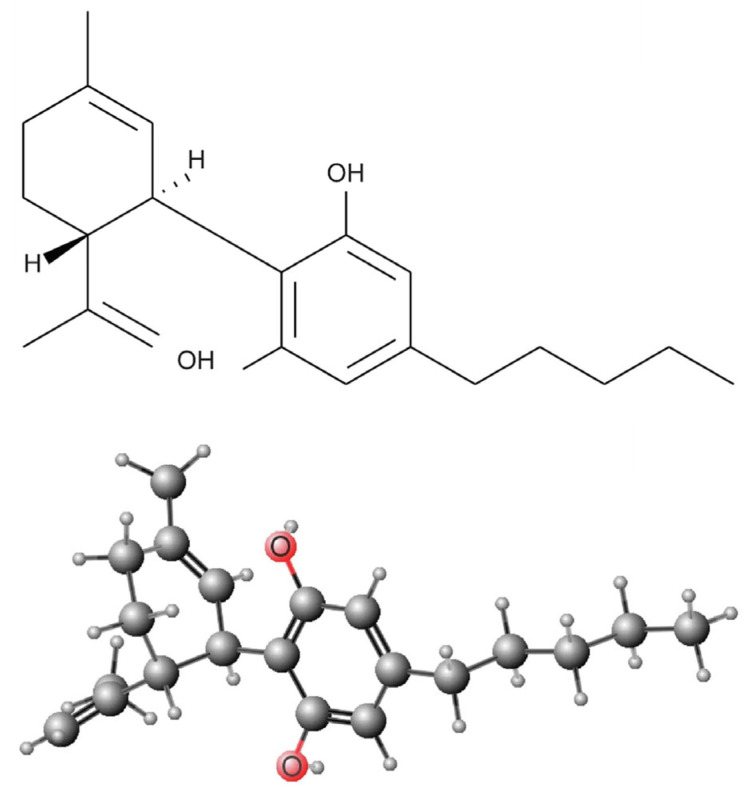
Chemical structure of CBD.

At present, an innovative strategy using cannabidiol (CBD) has been developed to effectively prevent liver injury. CBD, a fat-soluble non-psychoactive cannabinoid, was first extracted in the 1840s and its chemical structure was elucidated in 1963 [7] (Scheme 1). CBD has garnered increasing attention for its beneficial effects, including antioxidant, anti-inflammatory, immunomodulatory, antitumor, and neuroprotective beneficial effects [7]. Currently, some states in the United States have allowed the legal use of CBD in food [8]. The Canadian Health Food Association (CHFA) has highlighted on the positive economic impact of using CBD in non-human primates, food, and beverages on economic recovery [9]. In addition, in the European Union (EU), CBD was codified as a “novel food” in 2019 [10]. Notably, more and more studies have demonstrated the prominent role of CBD in protecting the liver. The results of Ma et al. demonstrated that CBD alleviated CCl_4_-induced hepatic fibrosis in mice by modulating the NF-κB and PPAR-α pathways [11]. Chen et al. systematically summarized the significant role and potential mechanisms of CBD in the treatment of different types of liver diseases, including non-alcoholic fatty liver disease, alcoholic liver disease, chemical liver injury, liver fibrosis, and liver cancer [12]. Given its therapeutic potential in the liver, CBD is a promising candidate for liver protection. However, its application in food and medicine is greatly limited due to its characteristics of easy light and temperature decomposition, poor water solubility, and low bioavailability [13].

In order to overcome these shortcomings, nano drug delivery systems were widely developed. Because of their unique nano-size, these nano drug delivery systems can effectively accumulate at the target site through EPR effects [14]. Notably, these nano drug co-delivery methods not only improve the stability and bioavailability of the drug and prolongs its duration of action in the body, but also achieve its controlled delivery and release and reduce the risk of adverse side effects, thus enhancing therapeutic efficacy [15]. However, traditional nano drug delivery systems are limited by their low targeting efficiency and poor therapeutic efficacy [16]. Hence, there is an urgent need for more effective disease treatment carriers with stronger disease site targeting and stimulus responsiveness, which can be accurately administered at the inflammatory site to minimize off-targeted side effects [17]. In particular, a new approach (targeted delivery system) has been proposed by integrating the properties of traditional drug carriers with biomaterials sensitive to various biochemical signals (pH, ROS, GSH, and enzymes), as well as surface functionalization of nanoparticles to bind specific target receptors [18].

Pullulan (PP) is a water-soluble neutral polysaccharide, which has been deemed safe by the U.S. FDA [19]. Pullulan, as a natural biomaterial, has the advantages of great biodegradability, biocompatibility, and non-toxicity, which have been widely studied and applied in the fields of food and biomedicine [15]. Particularly, pullulan, as a ligand of the asialoglycoprotein receptor (ASGPR), has inherent liver affinity, which is an ideal choice for the construction of liver-targeted nano drug carriers [20]. However, nanocarriers based on pullulan polysaccharides first need to be hydrophobic-modified to exhibit amphiphilic properties in water. Interestingly, as the main component of bile acids, deoxycholic acid (DA) contains carboxylic groups, which are ideal ligands for hydrophobic modification of polysaccharides [21]. Additionally, α-Lipoic acid (α-LA) is a naturally found short-chain fatty acid that includes disulfide bonds, and is also a natural powerful antioxidant that can be synthesized in the human body [22]. α-Lipoic acid (α-LA) exhibits diverse biological functions which can effectively treat cardiovascular and cerebrovascular diseases and diabetes [23]. Also, the disulfide bonds in the α-LA chemical structure can be broken under high concentrations of GSH to achieve rapid drug release, so it is commonly employed in designing nanocarriers with redox-responsive properties [24].

Based on the above principles, we attempted to construct a liver-targeted and GSH-response nano-delivery system using pullulan polysaccharide as the raw material. We hypothesized that the system constructed could effectively deliver CBD, thereby effectively preventing CCl_4_-induced ALI. Herein, we first grafted DA and α-LA onto PP to obtain DA-PP-LA, and further used polymers DA-PP-LA to construct nanoparticles for the effective delivery of CBD (CBD NPs). The particle size, potential, and microscopic morphology of CBD NPs and their protective effects on H_2_O_2_-induced liver injury were investigated. Finally, the mitigative effect of CBD NPs on CCl_4_-induced ALI was explored. This study offers a novel approach for developing a multifunctional delivery system that combines targeting and stimulus response to enhance CBD bioavailability and mitigate liver cell injury.

## 2. Materials and Methods

### 2.1. Materials

Deoxycholic acid (DA) was provided by Shanghai Yuanye Biological Technology Co., Ltd. (Shanghai, China). Pullulan (PP), dicyclohexylcarbodiimide (DCC), and α-lipoic acid (α-LA) were supplied by Macklin Biochemical Co., Ltd. (Shanghai, China). The chemical compound 4-(Dimethylamino)pyridine (DMAP) was acquired from Sigma-Aldrich Co. (St. Loius, MO, USA). Cannabidiol (CBD) and glutathione were supplied by Shanghai Aladdin Biochemical Technology Co., Ltd. (Shanghai, China). Inflammatory cytokines (TNF-α, IL-1β, and IL-10) were obtained from Shanghai enzyme Union Biotechnology Co., Ltd. (Shanghai, China). Mitochondrial membrane potential (MMP) and annexin V-FITC apoptosis detection kits were obtained from the Beyotime Institute of Biotechnology (Shanghai, China). Other related testing kits were purchased from the Nanjing Jiancheng Bioengineering Institute (Nanjing, China).

### 2.2. Synthesis of Polymer Deoxycholic Acid-Pullulan

The polymer deoxycholic acid-pullulan (DA-PP) was synthesized using the method of Xu et al. [25]. PP was dissolved in dried DMSO to obtain the PP solution (25 mg/mL). Meanwhile, a DMSO solution containing deoxycholic acid (DA, 1 g), DCC (1 g), and DMAP (0.6 g) was reacted for 2 h. Subsequently, the PP solution and DA solution were mixed and reacted for 48 h. The polymer solution after centrifugation is precipitated with alcohol and washed. The polymer DA-PP was collected.

### 2.3. Synthesis of Polymer Deoxycholic Acid-Pullulan-Lipoic Acid

The polymer deoxycholic acid-pullulan-lipoic acid was synthesized using the method of Xu et al. [25]. Firstly, the α-LA solution was treated with 0.5 g DCC and 0.4 g DMAP for 2 h. Subsequently, the polymer DA-PP solution (12.5 mg/mL) and the above LA solution were mixed and continuously reacted for 72 h. The polymer solution was precipitated and washed. The polymer DA-PP-LA was collected.

### 2.4. Structural Characterization of Polymers

Functional group changes in polymers were determined using an FT-IR spectrometer (Perkin Elmer, Waltham, MA, USA). The polymers were dissolved in DMSO-d6, and the ^1^H NMR of polymers was detected with a Bruker AVANCE III NMR spectrometer (Bruker, Fällanden, Switzerland). Additionally, the element distribution of polymer DA-PP and DA-PP-LA was measured through a X-Max 50 energy-dispersive spectrometer (Oxford Instruments, Abingdon, UK).

### 2.5. Preparation of DA-PP-LA NPs

Cannabidiol (CBD) loaded nanoparticles (CBD NPs) were prepared by a reverse solvent method according to the previous research with slight modifications [17]. In a nutshell, distilled water (2.5 mL) was dropped into DMSO containing DA-PP-LA (50 mg) and CBD (5 mg) and stirred for 2 h to obtain a CBD NP suspension. The suspension was then treated with ultrasonic energy for 10 min to make it uniformly dispersed. The dispersion of CBD NPs was dialyzed for 24 h. The dispersion was centrifuged, filtered, and lyophilized to obtain CBD NPs. Blank NPs were prepared using a similar program.

### 2.6. Characterization of CBD NPs

The particle size, polydispersity index, and zeta potential of Blank NPs and CBD NPs were determined by a Zetasizer Nano ZSE (Malvern Instruments Limited, Malvern, UK). The morphological characteristics of Blank NPs and CBD NPs were analyzed using transmission electron microscopy (TEM, JEM-2100F, JEOL, Akishima, Japan). The chemical structures were determined by an FT-IR spectrometer, a UV/VIS spectrometer (Lambda 35, Perkin Elmer, Waltham, MA, USA), and an X-ray diffractometer (XRD-7000, Shimadzu, Japan).

### 2.7. Determination of Encapsulation Efficiency and Drug Loading

The encapsulation efficiency (EE) and drug loading (DL) of CBD NPs were determined using HPLC (Agilent Technologies Inc., Santa Clara, CA, USA). The column was a C18 column (5 μm, 4.6 × 250 mm). Acetonitrile and water (65:35) were used as mobile phases. The flow rate was 1 mL/min.

Firstly, the standard curve was drawn. Then, CBD NPs were dispersed in acetonitrile and treated with ultrasonic energy for 30 min. The broken CBD NP solution was filtered and detected at the wavelength of 220 nm. The EE and DL of CBD were calculated as follows:

EE (%) = (The weight of CBD in nanoparticles)/(The weight of initial CBD) × 100%(1)

DL (%) = (The weight of CBD in nanoparticles)/(The weight of nanoparticles) × 100%(2)

### 2.8. In Vitro Release of CBD

The release behavior of CBD NPs was analyzed by dialysis. Different PBS buffer solutions (pH 7.4, pH 7.4 + 10 mM GSH) were used as release media to simulate different humoral environments. In brief, dialysis bags containing the CBD NP dispersion were placed in different release media at 37 °C and shaken. At appropriate intervals, the release medium was removed and promptly replenished with the same medium. The cumulative release content of CBD in different environments was measured with HPLC.

### 2.9. Cell Culture

Human hepatocellular carcinoma cells (HepG2 cells) were procured from Wuhan Procell Life Technology Co., Ltd. (Wuhan, China). A DMEM medium supplemented with 10% serum and 1% antibiotics was selected. The cell culture environment was 37 °C and contained 5% CO_2_.

### 2.10. Cellular Uptake

Cell experiments were categorized into free FITC, FITC NPs, and FITC NPs + GSH. HepG2 cells were seeded onto 6-well plates (density 10^6^ μL/well) and incubated for 24 h. The free FITC (0.5 μg/mL) or FITC NPs (FITC concentration equivalent of 0.5 μg/mL) were added into the wells for 3 h. For FITC NPs + GSH, the cells were pretreated with 10 mM of GSH for 1 h, followed by a 3 h incubation of FITC NPs. After washing twice with PBS, cells were treated with 4% paraformaldehyde and DAPI, respectively. Finally, the cells were sealed with a mixture of 1 mL glycerol and PBS (9:1). The cellular uptake behavior was recorded with a fluorescent inverted microscope (Nikon, Tokyo, Japan).

### 2.11. Apoptosis

HepG2 cells (10^6^ μL/well) with good growth status were exposed to CBD (0.5 μg/mL), CBD NPs (CBD concentration equivalent of 0.5 μg/mL), or CBD NPs + GSH (CBD concentration equivalent of 0.5 μg/mL) and cultured for 24 h. Cells were exposed to hydrogen peroxide (H_2_O_2_, final concentration: 0.6 mM) for 8 h. Cells were collected and processed with an apoptosis kit. Finally, apoptosis was evaluated by a flow cytometer (BD Biosciences, Franklin Lakes, NJ, USA), and the data were analyzed by FlowJo 10.8.1 software (Ashland, OR, USA).

### 2.12. Determination of ROS Level

HepG2 cells (10^6^ μL/well) with good growth status were co-incubated with CBD (0.5 μg/mL), CBD NPs (CBD concentration equivalent of 0.5 μg/mL), or CBD NPs + GSH (CBD concentration equivalent of 0.5 μg/mL), followed by treatment with H_2_O_2_ for 8 h. Cells were incubated with a culture medium containing probe DCFH-DA for 30 min. Cells were collected, washed, and added to a 96-well plate. The absorbance was recorded at an excitation wavelength of 485 nm and an emission wavelength of 525 nm using a fluorescence microplate.

### 2.13. Determination of Oxidative Stress Level

HepG2 cells (10^6^ μL/well) with good growth status were treated with CBD (0.5 μg/mL), CBD NPs (CBD concentration equivalent of 0.5 μg/mL), or CBD NPs + GSH (CBD concentration equivalent of 0.5 μg/mL) for 24 h, and then incubated with H_2_O_2_ for 8 h (final concentration: 0.6 mM). Then, the oxidative stress level of cells, including the SOD level, MDA content, LDH activity, and MPO activity, was evaluated with commercial kits.

### 2.14. Inflammation Level Analysis

HepG2 cells were seeded into 6-well plates (10^6^ μL/well) and incubated. After 24 h, the cells were treated with CBD (0.5 μg/mL), CBD NPs (CBD concentration equivalent of 0.5 μg/mL) or CBD NPs + GSH (CBD concentration equivalent of 0.5 μg/mL) for 24 h. Cells were exposed to H_2_O_2_ for 8 h. The content of inflammatory factors (NO, iNOS, TNF-α, IL-1β, ad IL-10) in cells was measured with commercial kits.

### 2.15. Determination of MMP Level

HepG2 cells were seeded into 6-well plates (10^6^ cells/well) for 24 h. Cells were co-cultured with a serum-free medium containing CBD (0.5 μg/mL), CBD NPs (CBD concentration equivalent of 0.5 μg/mL), or CBD NPs + GSH (CBD concentration equivalent of 0.5 μg/mL) for 24 h. The cells were then incubated with H_2_O_2_ for 8 h. Subsequently, the cells were dissociated with trypsin and centrifuged (1000 rpm, 10 min) for collection. Cells were processed through the MMP kit. The absorbance was detected using a fluorescence microplate analyzer at 485 nm, 535 nm and 535 nm, 595 nm, respectively.

### 2.16. Biodistribution In Vivo

To evaluate the liver-targeting ability of nanoparticles, Cy7 was used as a tracer to study the distribution of nanoparticles in vivo. First, nanoparticles loaded with Cy7 (Cy7 NPs) were prepared using similar methods to CBD NPs. Blab/c mice were orally given free Cy7 (dosage: 10 mg/kg) and Cy7 NPs (dosage: 10 mg/kg, the same Cy7 concentration equivalent). The mice were killed at different times and the distribution of free Cy7 and Cy7 NPs in the main organs of the mice was recorded by a live animal imager (American Molecular Devices Corporation, San Jose, CA, USA).

### 2.17. Alleviating Effect of CBD NPs on ALI

#### 2.17.1. Specific Protocols for Mouse Models of ALI

Male Balb/c mice (6–8 weeks old) were provided by Hunan Slake Jingda Experimental Animal Co., Ltd. (Changsha, China). All animal experimental procedures were approved by the Experimental Animal Health Ethics Committee of Hainan University (certificate number: HNUAUCC-2021-00046). All mice were housed in a pathogen-free, clean room with environmental conditions maintained at 21–25 °C and 50–55% relative humidity, under a 12 h dark/light cycle. They had unlimited access to sterilized basal feed and water. After 7 days of adaptive feeding, all mice were randomly distributed into six different groups (n = 10): control group, model group (CCl_4_), positive control group (silymarin, 50 mg/kg), CBD (10 mg/kg), CBD NPs (CBD equivalent of 10 mg/kg), and Blank NPs (equivalent of CBD NPs). Each mouse was numbered and received oral doses of the specified samples for 21 days. Next, all groups except for the control received intraperitoneal injections of a 0.2% CCl_4_ solution in olive oil (dose: 10 mL/kg).

#### 2.17.2. Determination of Biochemical Indicators

The mice were euthanized, blood was collected from the eyeball, and their livers were harvested and photographed. Next, part of the mouse liver was homogenized, and the levels of inflammatory markers (MPO, NO, iNOS, TNF-α, IL-1β, and IL-10) and CAT activity in the liver tissue of the mice were assessed. In addition, mouse serum was collected to measure the levels of liver function indicators (ALT, AST, and TBIL) and oxidative stress markers (SOD and MDA).

#### 2.17.3. Organizational Analysis

Lastly, a section of the liver was preserved in 4% paraformaldehyde and then subjected to H & E staining and immunohistochemical analysis using heme oxygenase-1 (HO-1) antibodies.

### 2.18. In Vivo Safety Analysis

Blab/c male mice (6–8 weeks old) were set into four groups (n = 10): PBS, CBD, Blank NPs, and CBD NPs. Each group received respective samples (10 mg/kg) for 21 days. The mice’s body weights were recorded every seven days. At the end of this period, blood was drawn and centrifuged (3500 rpm, 10 min) to extract serum. The serum levels of ALT, AST, CRE, and BUN were measured. The main organs were harvested for H & E staining.

### 2.19. Statistical Analysis

Each experiment was performed in triplicate and the results were then reported as averages and standard deviations of these measurements. The data were analyzed by a one-way analysis of variance (ANOVA) and a post hoc multiple comparison paired Tukey test. The data were considered statistically significant if *p* < 0.05.

## 3. Results

### 3.1. Synthesis and Characterization of Polymers

**Figure 1:**
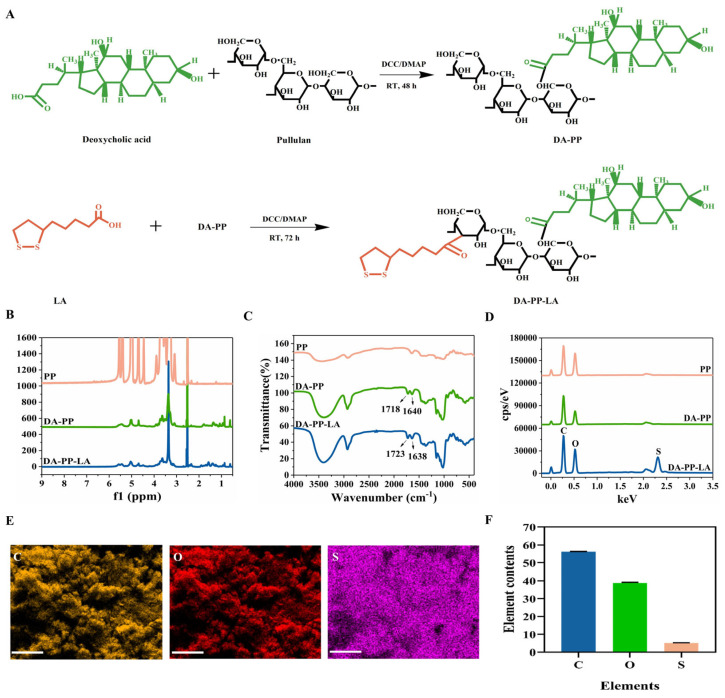
Synthesis and structural characterization of polymer DA-PP-LA. (**A**) Synthesis process of polymer DA-PP-LA. (**B**) ^1^H NMR spectra, (**C**) infrared spectra, and (**D**) X-ray energy spectra of pullulan polysaccharides, polymers DA-PP and DA-PP-LA. (**E**) Element distribution images and (**F**) element content of polymer DA-PP-LA. Scale bar = 50 nm. Values are presented as mean ± SD (n = 3).

Firstly, the polymer DA-PP was synthesized. The carboxyl group of DA was activated and coupled with PP by forming an ester bond to form the polymer DA-PP. Next, α-LA and the polymer DA-PP were further synthesized by an esterification reaction to obtain the polymer DA-PP-LA (Figure 1A). The structures of pullulan polysaccharide, the polymer DA-PP, and the polymer DA-PP-LA were analyzed by ^1^H NMR. Compared with the ^1^H NMR of PP, new characteristic peaks of 0.63–1.80 ppm appeared in the ^1^H NMR of DA-PP (Figure 1B and Figure S1–S3). Among them, the signals of 0.63 ppm, 0.86 ppm, and 0.94 ppm were attributed to 18-CH_3_, 19-CH_3,_ and 21-CH_3_ of DA, respectively [26,27]. In addition, the signal of 1.0–1.8 ppm corresponded to the methylene-methine envelope of DA. The above results proved that the DA-PP polymer was successfully synthesized [27]. In the ^1^H NMR of the polymer DA-PP-LA, the characteristic peaks of 1.39–1.70 ppm, 1.86–1.92 ppm, 2.35 ppm, 2.41–2.46 ppm and 3.12–3.23 ppm were attributed to the existence of CH_2_CH_2_CH_2_-lipoic ring, SSCH_2_CH_2_CH, CH_2_COO, SSCH_2_CH_2_CH, and SSCH_2_CH_2_CH signals in α-LA, respectively [28], indicating the successful connection between LA and the polymer DA-PP. Furthermore, in order to further verify the successful synthesis of polymers, the structures of PP, the polymer DA-PP, and the polymer DA-PP-LA were further analyzed by infrared spectroscopy. As shown in Figure 1C and Figure S4–S6, compared with PP, the polymers DA-PP and DA-PP-LA showed characteristic absorption peaks of ester bonds at 1718 cm^−1^ and 1723 cm^−1^, indicating that both DA and LA were successfully linked to PP. Also, the element distribution and element content of PP, the polymer DA-PP, and the polymer DA-PP-LA were determined. As shown in Figure 1D–F, the polymer DA-PP-LA contained C, O, and S elements with contents of 56.19 ± 0.14%, 38.79 ± 0.41%, and 5.03 ± 0.28%, respectively. All the results confirmed the effective coupling of the polymer DA-PP and the polymer DA-PP-LA.

### 3.2. Structural Analysis of CBD NPs

**Figure 2:**
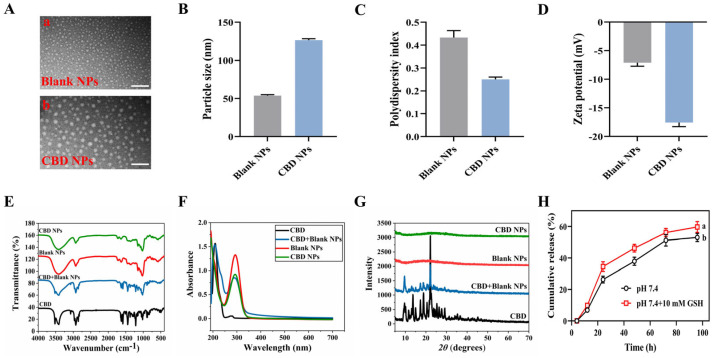
Structural analysis of CBD NPs. (**A**) TEM images of (**a**) Blank NPs, and (**b**) CBD NPs. The scale bar = 200 nm. (**B**) The particle size, (**C**) polydispersity index, and (**D**) zeta potential. (**E**) Infrared spectra, (**F**) ultraviolet spectra, and (**G**) X-ray diffraction pattern. (**H**) In vitro drug release behavior. The values are presented as the mean ± SD (n = 3). ^a,b^ Different letters represent significant differences (*p* < 0.05).

Using the polymer DA-PP-LA as the raw material, Blank NPs and CBD NPs were prepared through self-assembly. TEM images revealed that NPs were uniformly spherical and dispersed evenly in water (Figure 2A). Compared to Blank NPs (53.87 ± 1.00 nm), the size of CBD NPs had significantly increased (126.5 ± 1.56 nm), which may be due to the larger hydrophobic core volume caused by CBD interception (Figure 2B). Additionally, the PDIs of Blank NPs and CBD NPs were 0.43 ± 0.02 and 0.25 ± 0.01, indicating that they had a relatively narrow size distribution (Figure 2C). Furthermore, the zeta potentials measured were −7.09 ± 0.50 mV for Blank NPs and −17.57 ± 0.57 mV for CBD NPs (Figure 2D). The successful encapsulation of CBD into nanoparticles was confirmed by evaluating the infrared spectra. The absorption peaks of CBD were 3522 cm^−1^, 3413 cm^−1^, 2856 cm^−1^, 1583 cm^−1^, 1215 cm^−1^, etc. (Figure 2E). Interestingly, the absorption peaks noted earlier were also present in the spectra of the physical mixture of CBD and Blank NPs. However, there was an absence of a characteristic peak of CBD in the CBD NP spectra. Notably, the characteristic peaks of ester bonds appeared in both Blank NPs and CBD NPs at 1718 cm^−1^ and 1722 cm^−1^, which strongly indicated that both CBD-wrapped NPs and Blank NPs remained stable and did not decompose during the experiment. In the ultraviolet spectrum, the distinct peaks of CBD were identified as a strong absorption at 209 nm and a weaker one at 274 nm (Figure 2F). Clearly, the characteristic peaks were visible in the physical mixture of CBD and Blank NPs. Notably, the ultraviolet spectrum of CBD NPs resembled that of Blank NPs, lacking any absorption peaks of CBD. Furthermore, the XRD patterns showed similar results (Figure 2G). These findings suggest that CBD was embedded within the nanoparticles. Furthermore, the EE and DL of CBD NPs were 78.25 ± 5.04% and 7.11 ± 0.46%, respectively.

### 3.3. Evaluation of In Vitro Release Behavior

As shown in Figure 2H, the release of CBD increased over time. Notably, the CBD release at a pH of 7.4 with 10 mM GSH was higher than at a pH of 7.4 alone. After 96 h, the CBD release was 52.98 ± 2.58% in the pH of 7.4 group and 59.66 ± 3.46% in the pH of 7.4 + 10 mM GSH group. This result may be attributed to the disintegration of CBD NPs caused by GSH, indicating that CBD NPs had redox responsiveness. These results indicate that CBD NPs are an effective drug delivery system, capable of enhanced release at inflammatory or tumor sites with elevated GSH concentrations.

### 3.4. Cellular Uptake of Nanoparticles

**Figure 3:**
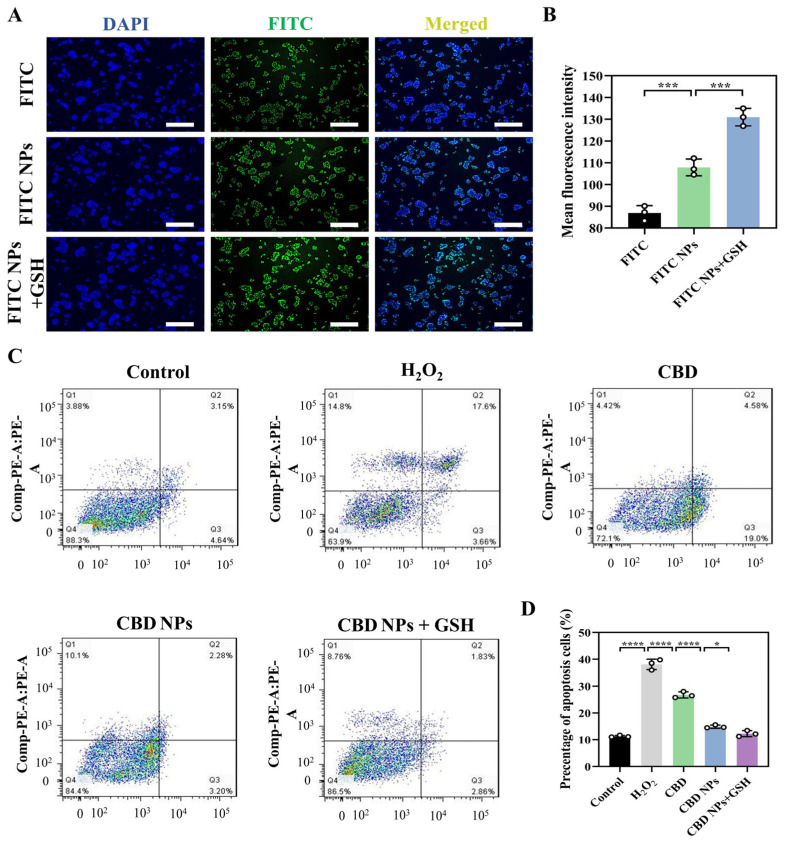
Determination of intracellular uptake and apoptosis of nanoparticles. (**A**) Cell uptake images and (**B**) average fluorescence intensity of HepG2 cells. Magnification: 4 times. Scale bar = 320 μm. (**C**) Flow cytometry analysis and (**D**) apoptosis percentage of HepG2 cells. Values are presented as mean ± SD (n = 3). Statistical significance is indicated as follows: * *p* < 0.05, *** *p* < 0.001, and **** *p* < 0.0001.

To examine the specific affinity of CBD NPs for HepG2 cells, nanoparticles loaded with FITC (FITC NPs) were prepared using the same method as for the preparation of CBD NPs. The uptake and cell distribution of nanoparticles in HepG2 cells were measured by a fluorescence inversion microscope. The fluorescence intensity of FITC NPs exceeded that of free FITC, suggesting that FITC NPs are more readily internalized by HepG2 cells than free FITC (Figure 3A,B). The reason for this phenomenon may be based on the fact that CBD NPs had excellent liver cell-specific affinity, thus promoting cell uptake. This result was confirmed by in vivo biodistribution experiments. Many studies have shown that pullulan can specifically bind to the asialoglycoprotein receptor (ASGPR) overexpressed on the surface of hepatocytes and can be effectively internalized by receptor-mediated endocytosis, thus showing good liver targeting ability [19]. Notably, after a 2 h pretreatment with GSH, more FITC NPs were absorbed into the HepG2 cells, resulting in stronger fluorescence intensity, which may be due to the better redox responsiveness of CBD NPs, which led to the degradation of nanoparticles and the rapid release of CBD. The results demonstrated that the prepared nanoparticles exhibited excellent cellular uptake capabilities.

### 3.5. Apoptosis Evaluation

The HepG2 cells were labeled with Annexin V-FITC and PI, and apoptosis was assessed by flow cytometry (Figure 3C,D). The results showed that H_2_O_2_ could significantly induce early apoptosis, late apoptosis, and even death in HepG2 cells (*p* < 0.05), with an apoptosis rate of 38.14 ± 1.55%. Interestingly, CBD and CBD NPs effectively protected cells from apoptosis. In addition, after GSH pretreatment, the apoptosis rate of HepG2 cells was significantly reduced (12.27 ± 0.91%). The results indicated that CBD NPs effectively shielded cells from damage and apoptosis induced by H_2_O_2_.

### 3.6. Protective Effect of CBD NPs against Cellular Oxidative Injury

**Figure 4:**
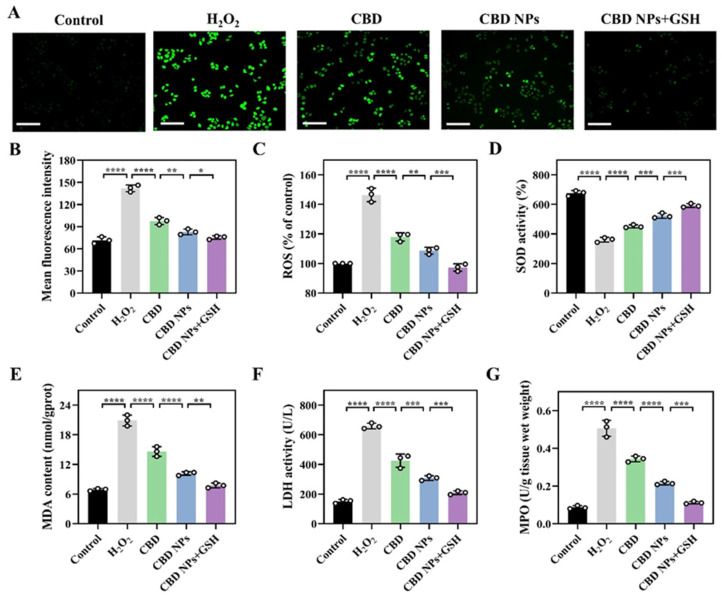
The alleviating effect of CBD NPs on oxidative stress and inflammation in HepG2 cells. (**A**) ROS fluorescence images, (**B**) mean fluorescence intensity, and (**C**) intracellular ROS level in HepG2 cells. Magnification: 10 times. The scale bar = 120 μm. The levels of (**D**) SOD, (**E**) MDA, (**F**) LDH, and (**G**) MPO in HepG2 cells. The values are presented as the mean ± SD (n = 3). Statistical significance is indicated as follows: * *p* < 0.05, ** *p* < 0.01, *** *p* < 0.001, and **** *p* < 0.0001.

Research indicates that oxidative stress plays a crucial role in hepatocyte damage, leading to inflammation and impaired mitochondrial function in liver cells [29]. Therefore, firstly, the level of ROS in HepG2 cells was measured. It is well known that DCFH-DA fluorescent probes are oxidized by overexpressed ROS to 2′,7′-dichlorofluorescein (DCF), showing green fluorescence. Therefore, the level of ROS in cells was detected by a DCFH-DA fluorescence probe. The level of ROS was overexpressed in HepG2 cells and showed strong green fluorescence after H_2_O_2_ treatment (Figure 4A,B). A significant reduction in green fluorescence within the cells in the CBD group and the CBD NPs group was observed, suggesting a decrease in oxidative stress. Oxidative stress levels were notably lower with CBD NPs compared to free CBD, likely due to the nanocarriers enhancing CBD uptake efficiently. This outcome aligned with the cell uptake results. Additionally, following GSH pretreatment, the fluorescence intensity of cells approached that of the control group. This result is likely due to the high intracellular GSH concentration breaking the disulfide bonds in CBD NPs, thereby accelerating nanoparticle degradation and CBD release. This result was proved in the drug release experiment in vitro. This result also proved the redox responsiveness of CBD NPs. Furthermore, the level of ROS in HepG2 cells was determined. Of note, consistent results were obtained (Figure 4C). Also, CBD and CBD NPs significantly reversed the decrease in cell SOD levels and the increase in MDA levels caused by H_2_O_2_ (Figure 4D,E). Moreover, the antioxidant level of cells treated with CBD NPs was significantly higher than that of free CBD. The excellent antioxidant ability proved that CBD NPs had great application potential in protecting liver cells from oxidative damage.

### 3.7. LDH Level Analysis

Research has demonstrated that LDH is normally found in the cytoplasm and cannot cross the cell membrane. However, when liver cells are damaged, increased membrane permeability causes LDH to be released from the cells [30]. To assess cell membrane integrity, the level of LDH release was measured. The findings indicated that H_2_O_2_ treatment significantly elevated LDH levels in HepG2 cells, indicating that HepG2 cells were injured, resulting in the destruction of the cell membrane (Figure 4F). Interestingly, CBD and CBD NPs inhibited the release of LDH in cells. Notably, LDH levels in cells of the CBD NPs + GSH group were significantly lower than those treated with CBD NPs alone. This reduction is likely due to the disulfide bond breaking and the rapid release of CBD, which further protects cells from damage. These findings demonstrate that CBD NPs effectively safeguard cells, thereby preserving cell membrane integrity.

### 3.8. Effect of Nanoparticles on the Activity of MPO

Moreover, after injury or inflammation, the level of MPO in cells will also increase excessively [31]. Therefore, the activity of MPO in HepG2 cells after different treatments was further determined. As anticipated, CBD and CBD NPs significantly inhibited the H_2_O_2_-induced increase in MPO activity. This result may result from high intracellular GSH concentrations breaking the disulfide bonds, accelerating nanoparticle degradation and CBD release (Figure 4G). This result was consistent with the results of drug release in vitro. The above results prove that CBD NPs could protect liver cells from injury by reducing the level of intracellular oxidative stress and inflammation.

### 3.9. Intervention Effect of CBD NPs on Liver Inflammation

**Figure 5:**
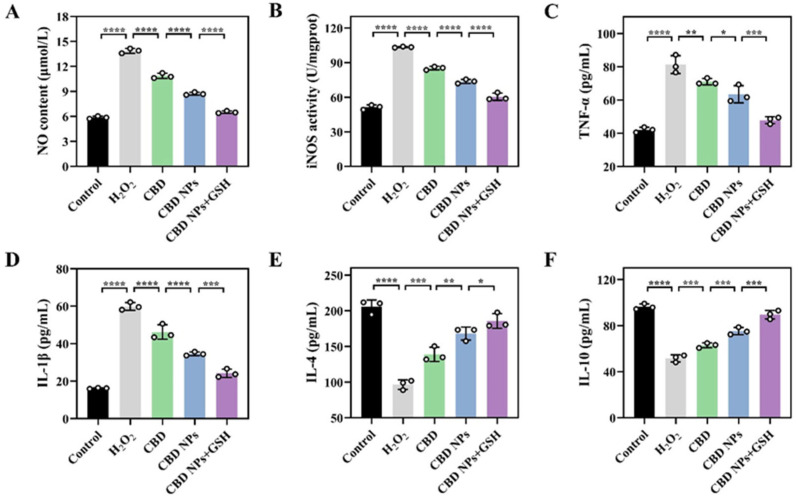
Inflammation level analysis. The levels of (**A**) NO, (**B**) iNOS, (**C**) TNF-α, (**D**) IL-1β, (**E**) IL-4, and (**F**) IL-10 in HepG2 cells. The values are presented as the mean ± SD (n = 3). Statistical significance is indicated as follows: * *p* < 0.05, ** *p* < 0.01, *** *p* < 0.001, and **** *p* < 0.0001.

Research has shown that oxidative stress can damage liver cells, leading to inflammation [32]. As shown in Figure 5A–F, H_2_O_2_ treatment significantly increased NO, iNOS, TNF-α, and IL-1β levels, while significantly decreasing IL-4 and IL-10 levels in HepG2 cells. In stark contrast to these findings, CBD and CBD NPs significantly improved the situation. Compared to the other two, CBD NPs had stronger anti-inflammatory effects, likely due to the enhanced internalization facilitated by the nanocarrier. Furthermore, CBD NPs + GSH exhibited even more significant anti-inflammatory activity compared to CBD and CBD NPs alone.

### 3.10. MMP Level Analysis

**Figure 6:**
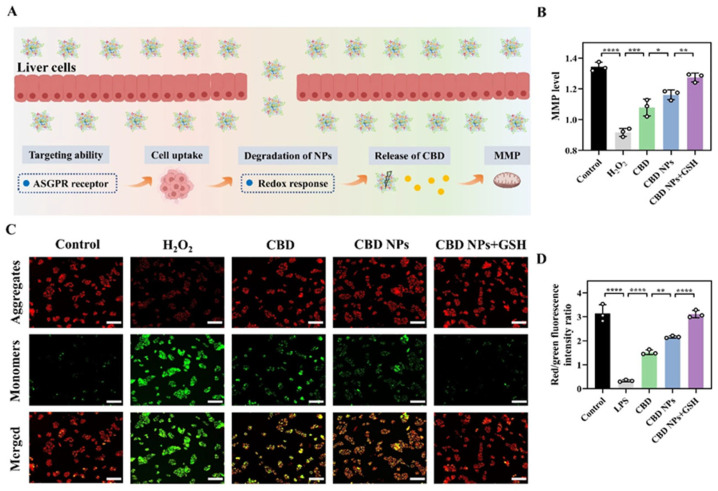
MMP level analysis. (**A**) A flow chart of the protective effect of nanoparticles on MMP. (**B**) The levels of MMP in HepG2 cells treated with different samples. (**C**) The fluorescence images of MMP and (**D**) the red/green fluorescence intensity ratio. Magnification: 10 times. The scale bar = 120 μm. The values are presented as the mean ± SD (n = 3). Statistical significance is indicated as follows: * *p* < 0.05, ** *p* < 0.01, *** *p* < 0.001, and **** *p* < 0.0001.

Cell injury is known to cause a decrease in MMP levels, which can be detected using the JC-1 fluorescence probe [33]. Consequently, the impact of nanoparticles on MMP in HepG2 cells was assessed with the JC-1 probe (Figure 6A). H_2_O_2_ treatment resulted in strong green fluorescence, suggesting the obvious destruction of MMP in cells (Figure 6C). Following treatment with different samples, there was a sequential increase in red fluorescence and a decrease in green fluorescence, resulting in a gradual rise in the red/green fluorescence intensity ratio (Figure 6D). This indicates that all treatments significantly preserved MMP balance in cells. Additionally, MMP levels were measured, revealing that MMP in HepG2 cells was disrupted after H_2_O_2_ treatment (Figure 6B). Both CBD and CBD NP treatments reversed MMP depolarization and early cell apoptosis. Furthermore, the CBD NPs + GSH group exhibited a significantly higher MMP compared to those treated with CBD NPs alone. These findings demonstrated that CBD NPs effectively maintain MMP balance, thus protecting mitochondria.

### 3.11. Determination of Biodistribution In Vivo

**Figure 7:**
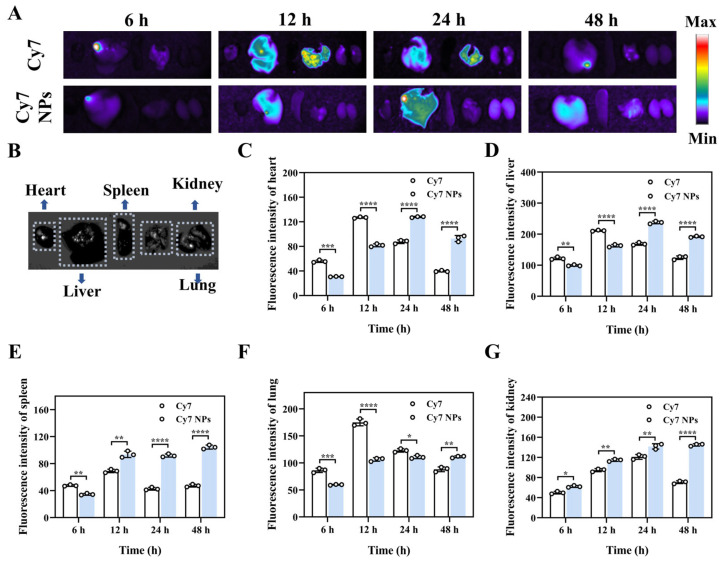
The biodistribution of nanoparticles in vivo. (**A**) Fluorescence images of the main organs. (**B**) Bright-field images of the main organs. The average fluorescence intensity of the (**C**) heart, (**D**) liver, (**E**) spleen, (**F**) lungs, and (**G**) kidneys of mice. The values are presented as the mean ± SD (n = 3). Statistical significance is indicated as follows: * *p* < 0.05, ** *p* < 0.01, *** *p* < 0.001, and **** *p* < 0.0001.

Figure 7B presents the bright field image of the main organs. As shown in Figure 7A, both free Cy7 and Cy7 NPs exhibited strong fluorescence signals in the liver, with the fluorescence signal of Cy7 peaking at 12 h. With the increase in time, the fluorescence intensity of free Cy7 in the liver gradually decreased, with only weak fluorescence observed at 48 h, indicating rapid clearance from systemic circulation. Interestingly, Cy7 NPs maintained tangible fluorescence intensity at 48 h, suggesting that Cy7 NPs extended the retention time of Cy7, delayed their clearance, and effectively improved their bioavailability. In addition to having a strong fluorescence intensity in the liver, the strong fluorescence signal of free Cy7 in the lungs was clearly observed, indicating that free drugs did not have liver targeting ability and could accumulate or be excreted in other organs. Compared to other organs, Cy7 NPs exhibited the strongest fluorescence signals in the liver. This indicated that the Cy7 NPs primarily accumulated in the liver and demonstrated excellent liver-targeting capabilities. The quantitative results of fluorescence intensity in main organs effectively validated the above conclusions (Figure 7C–G). These findings suggest that the nanocarrier developed can effectively reach liver tissue, enabling targeted drug delivery.

### 3.12. Alleviating Effect of CBD NPs on CCl_4_-Induced ALI

#### 3.12.1. The Effect of CBD NPs on Liver Function Indicators in ALI Mice

**Figure 8:**
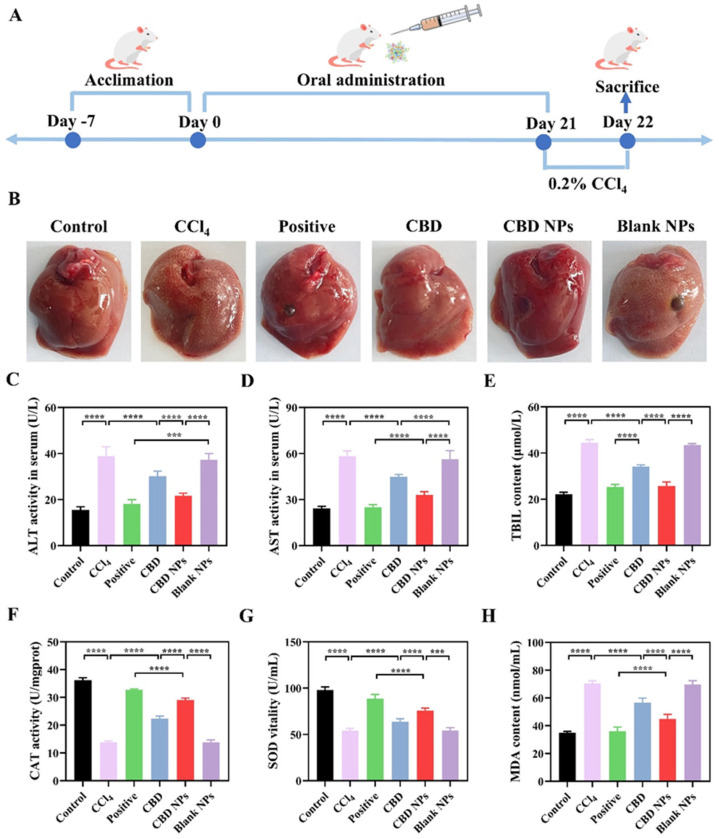
The alleviating effect of CBD NPs on CCl_4_-induced ALI. (**A**) The animal experimental protocol. (**B**) Liver images. The effects of CBD NPs on the levels of (**C**) ALT, (**D**) AST, (**E**) TBIL, (**F**) CAT, (**G**) SOD, and (**H**) MDA in the liver tissue of ALI mice. The values are presented as the mean ± SD (n = 3). Statistical significance is indicated as follows: *** *p* < 0.001, and **** *p* < 0.0001.

The animal experiment protocol is shown in Figure 8A. The liver in the healthy group appeared dark red with a very smooth surface. In contrast, after CCl_4_ induction, the liver of the model group mice turned light red and exhibited noticeable surface fibrosis (Figure 8B). The treatment of CBD and CBD NPs effectively alleviated CCl_4_-induced liver injury. Research has shown that levels of ALT, AST, and TBIL, key biomarkers of liver injury, increased significantly after CCl_4_ induction, indicating severe liver damage in ALI mice [34] (Figure 8C–E). Conversely, treatment with the CBD group and the CBD NPs group significantly reduced these biomarker levels. Notably, CBD NPs provided more useful liver protection, likely due to their ability to target delivery to liver tissue. This enhanced hepatoprotective effect is likely due to the targeted delivery of CBD to liver tissue by CBD NPs. The redox response facilitated the degradation of CBD NPs and the rapid release of CBD. Additionally, the serum levels of ALT, AST, and TBIL in Blank NPs-treated ALI mice did not differ significantly from the model group (*p* > 0.01), indicating no substantial impact on CCl_4_-induced ALI.

#### 3.12.2. The Effect of CBD NPs on Oxidative Stress Indicators in Serum of ALI Mice

Reports indicate that severe oxidative stress is a key factor in the pathogenesis of CCl_4_-induced ALI [35]. Thus, mitigating oxidative stress levels can effectively improve ALI. Notably, CBD NPs significantly increased CAT and SOD levels while decreasing MDA levels in ALI mice (Figure 8F–H). These findings suggest that CBD NPs protect the liver by enhancing its antioxidant capacity in ALI mice.

#### 3.12.3. The Effect of CBD NPs on the Level of Inflammatory Markers in the Liver of ALI Mice

**Figure 9:**
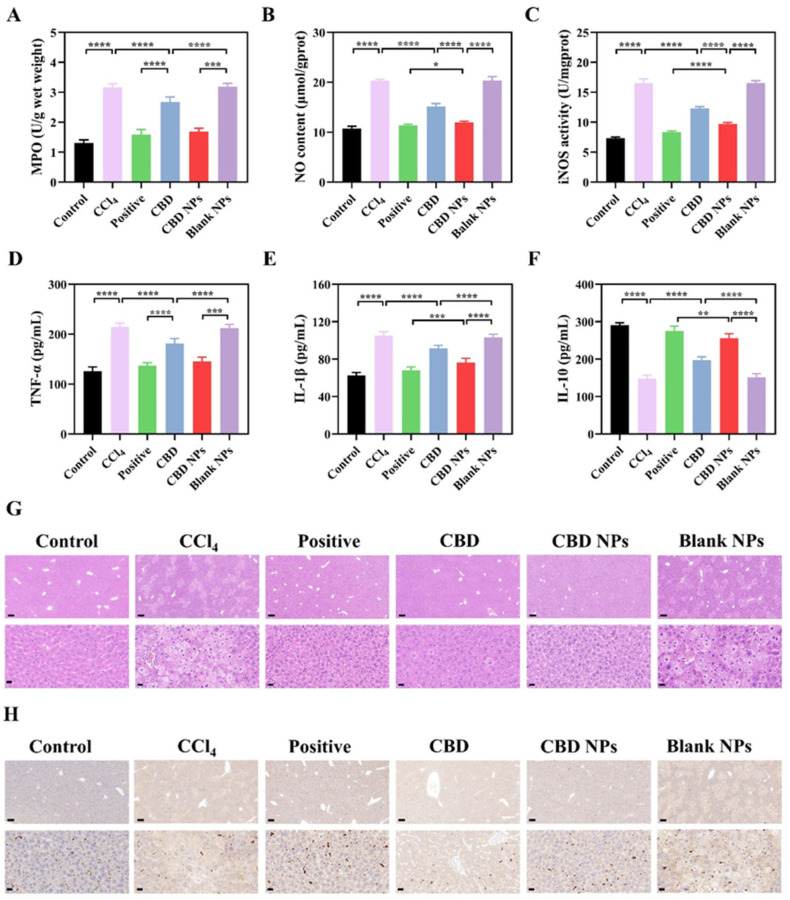
Mitigating effects of CBD NPs on inflammation in liver tissue of ALI mice. Effects of CBD NPs on (**A**) MPO, (**B**) NO, (**C**) iNOS, (**D**) TNF-α, (**E**) IL-1β, and (**F**) IL-10 levels. (**G**) H & E staining analysis. (**H**) Immunohistochemical analysis. Values are presented as mean ± SD (n = 3). Statistical significance is indicated as follows: * *p* < 0.05, ** *p* < 0.01, *** *p* < 0.001, and **** *p* < 0.0001.

Additionally, the levels of inflammatory markers in ALI mice were assessed. The results indicate that CCl_4_ induction leads to an increase in pro-inflammatory cytokines (MPO, NO, iNOS, TNF-α, and IL-1β) and a significant decrease in the anti-inflammatory cytokine IL-10, suggesting substantial liver inflammation (Figure 9A–F). As anticipated, CBD NPs significantly reverse this trend, demonstrating their effectiveness in reducing liver inflammation in ALI mice.

#### 3.12.4. Organizational Analysis

In addition, the liver-protective effect of CBD NPs was evaluated through H & E staining. The normal livers exhibited a neatly arranged liver cell structure and regular morphology. (Figure 9G). The induction of CCl_4_ caused serious liver damage in the model group, including hepatocyte disorder, hepatocyte vacuolation, and fibrosis. CBD NPs provided effective protection against CCl_4_-induced ALI. Furthermore, the level of HO-1 in the liver tissue was assessed through immunohistochemistry. The findings revealed that CBD NPs significantly increased HO-1 expression, thereby alleviating CCl_4_-induced ALI (Figure 9H).

### 3.13. In Vivo Biosafety Analysis

**Figure 10:**
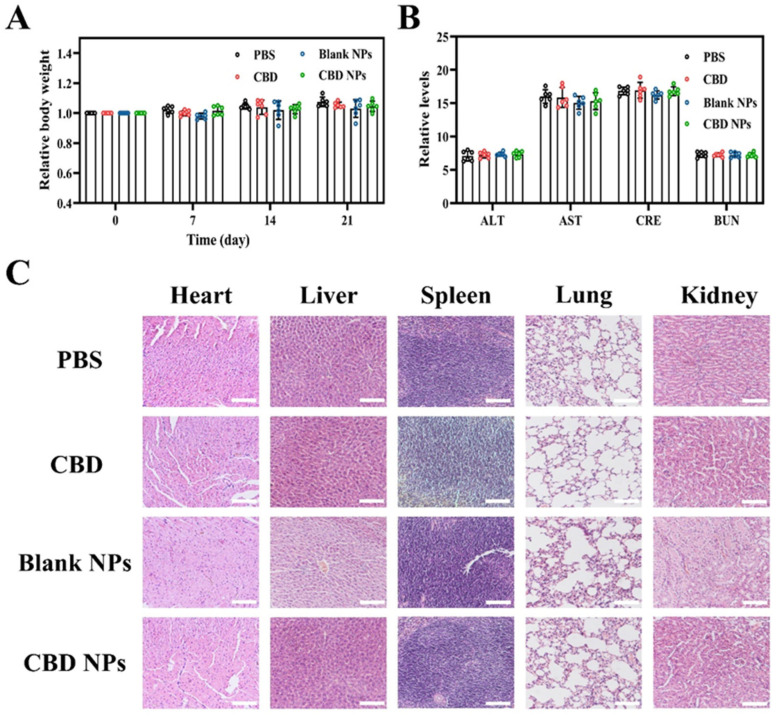
In vivo safety of nanoparticles. Changes in (**A**) body weight, (**B**) liver, and kidney function indexes, and (**C**) H & E staining of major organs in mice treated with PBS, CBD, Blank NPs, and CBD NPs. Magnification: 10 times. Scale bar = 100 µm. Values are presented as mean ± SD (n = 6).

Given their excellent alleviating effect on liver injury, the in vivo biological safety of the nanoparticles was evaluated through histological analysis. As anticipated, there were no significant differences in body weight or levels of liver and kidney function indicators (ALT, AST, CRE, and BUN) among the different groups of samples compared to the PBS group (Figure 10A,B). Additionally, H & E staining of major organs (heart, liver, spleen, lung, and kidney) revealed no significant histological damage, such as necrosis, hyperemia, or bleeding (Figure 10C). These findings indicate that CBD, Blank NPs, and CBD NPs possess good biological safety, supporting their potential for practical medical and clinical application.

## 4. Discussion

Despite significant progress in the treatment of ALI over the past decades, there is still a need to develop safe and effective strategies to effectively inhibit the progression of ALI. Studies have shown that CBD plays a beneficial role in protecting the liver. The results of Mukhopadhyay et al. [36] demonstrated that CBD protected against hepatic ischemia/reperfusion injury by attenuating inflammatory signaling and responses, oxidative/nitrative stress, and cell death. Similar results were obtained by Fouad et al. [37]. However, the stability of CBD is affected by a variety of factors, as evidenced by its susceptibility to degradation under light, temperature, and pH, leading to its low bioavailability. Based on this, in order to improve the stability and bioavailability of CBD, in this study, a nano-delivery system was successfully constructed by grafting deoxycholic acid (DA) and α-lipoic acid (α-LA) with pullulan polysaccharides, respectively. The results demonstrated that CBD NPs exhibited excellent liver targeting ability. They could significantly accumulate in the liver and increase their circulation time in the liver, thereby enhancing the bioavailability of CBD. In addition, due to their redox-responsive properties, CBD NPs achieved rapid CBD release under conditions of high GSH concentration at the site of hepatic inflammation, resulting in the effective protection of hepatic function, an improvement of oxidative stress levels, the inhibition of inflammatory factor production, and the protection of the liver from histological damage, leading to effective prevention of CCl_4_-induced ALI. However, in this study, the targeting ability of CBD NPs was evaluated only by in vivo biodistribution, and the redox response properties of CBD NPs were analyzed only by cellular experiments, whereas the specific hepatic targeting mechanism and the specific redox response mechanism of CBD NPs are unknown and need to be further explored.

After ensuring that CBD NPs can target the liver and achieve specific release, efficient cellular uptake is key for them to exert beneficial effects in CCl_4_-induced ALI. It has been shown that smaller-sized (<500 nm) nanoparticles are more likely to penetrate biological barriers, promote their cellular uptake, and enhance their physiological functions [38]. Zhang et al. [39] prepared *Cordyceps sinensis* exopolysaccharide-selenium nanoparticles (EPS-SeNPs) with different particle sizes for inducing apoptosis in HepG2 cells. The results demonstrated that the nanoparticles with the smallest particle size had the strongest anti-tumor activity. Guo et al. [40] explored the lymph node targeting ability of β-glucan modified nanoparticles with different particle sizes. The results showed that nanoparticles with a smaller particle size had stronger lymphatic targeting efficiency and a stronger ability to promote dendritic cell maturation. In this study, we found that both particle size and TEM results showed that the particle size of CBD NPs (126.5 ± 1.56 nm) was much smaller than previously reported results, such as nanostructured lipid carriers loaded with CBD (177 ± 3 nm) [41] and polylactic acid co glycolic acid (PLGA) nanoparticles loaded with CBD (171 nm) [42], and showed stronger hepatoprotective activity, further confirming the above viewpoint. However, in addition to the important effect of particle size, the cellular uptake pathway of CBD NPs is also critical for their cellular uptake efficiency and activity exertion.

As a hepatotoxin that has been used to induce ALI, CCl_4_ is metabolized in vivo to a variety of free radicals, such as CCl3·, CCl3OO·, and ROS [43]. These free radicals bind to polyunsaturated fatty acids, leading to oxidative stress and further liver damage. This implies that oxidative stress plays a key role in CCl_4_-induced ALI. Therefore, the changes induced by CBD NPs on oxidative stress indicators in serum and inflammatory mediator levels in liver tissues of ALI mice were measured at the animal level, respectively. Therefore, the changes of CBD NPs on oxidative stress indicators (CAT, SOD, and MDA) in serum and inflammatory cytokine levels (MPO, NO, iNOS, TNF-α, and IL-1β) in liver tissues of ALI mice were determined at the animal level, respectively. The expression of the antioxidant factor HO-1 was also explored by immunohistochemistry. The results strongly demonstrated the prominent role of CBD NPs in maintaining the balance of oxidative stress levels and reducing inflammation in ALI mice. However, due to the limitations of many conditions, the molecular regulatory mechanism of CBD NPs in alleviating CCl_4_-induced ALI has not yet been elucidated and needs further research in the future.

## 5. Conclusions

In this study, a liver-targeted nanoparticle delivery system based on the redox response was developed to target the delivery of CBD to the liver for the effective prevention of ALI. CBD NPs showed good biocompatibility as well as in vivo safety. Interestingly, CBD NPs exhibited excellent liver targeting ability and redox response characteristics, effectively promoting the internalization of CBD into HepG2 cells. More importantly, CBD NPs showed great therapeutic potential in CCl_4_-induced ALI, as evidenced by the effective protection of liver function, the improvement of oxidative stress levels, the inhibition of inflammatory factor production, and the protection of the liver from histological damage. In summary, the nano-delivery system prepared in this study not only improved the bioavailability of CBD but also achieved targeted delivery and specific release of CBD, providing a potential platform for effective intervention in ALI. Nevertheless, the hepatic targeting mechanisms, cellular uptake pathways, redox response mechanisms, and molecular regulatory mechanisms of CBD NPs for the effective alleviation of ALI need to be further explored to expand the application of CBD NPs in potential therapeutic applications.

## Data Availability

The data presented in this study are available on request from the corresponding author. The data are not publicly available as the first author needs to apply for a doctoral degree.

## Supplementary Materials

The following supporting information can be downloaded at: https://www.mdpi.com/article/10.3390/foods13152464/s1, Figure S1: ^1^H NMR spectrum of pullulan polysaccharides; Figure S2: ^1^H NMR spectrum of polymer DA-PP; Figure S3: ^1^H NMR spectrum of polymer DA-PP-LA. Figure S4: Infrared spectrum of pullulan polysaccharides; Figure S5: Infrared spectrum of polymer DA-PP; Figure S6: Infrared spectrum of polymer DA-PP-LA.
