# Supplementary material for: Liver-Targeted Nanoparticles Loaded with Cannabidiol Based on Redox Response for Effective Alleviation of Acute Liver Injury
Source: Foods. 2024 Aug 4;13(15):2464. doi: 10.3390/foods13152464 (PMC11311329; doi:10.3390/foods13152464)
Supplement: Supplementary file 1 [file foods-13-02464-s001.zip › foods-3077007-supplementary.pdf]

Supplementary Material

Liver-targeted nanoparticles loaded with cannabidiol based on redox response for effective alleviation of acute liver injury

Xuan Zhang [a, c, d], Xiangzhou Yi [a, c, d], Xia Gao [a, c, d], Yongcheng Li [a, c, d], Xuanri Shen [a, b, c, d *]

[a] School of Food Science and Engineering, Hainan University, Haikou, 570228, China

[b] College of Food Science and Engineering, Hainan Tropical Ocean University, Sanya, 572022, China

[c] Key Laboratory of Food Nutrition and Functional Food of Hainan Province, Haikou, 570228, China

[d] Hainan Engineering Research Center of Aquatic Resources Efficient Utilization in South China Sea, Hainan University, Haikou, 570228, China

*** To the Correspondence**

Name: Xuanri Shen

E-mail: shenxuanri2009@163.com

Mailing address: College of Food Science and Engineering, Hainan University, No. 58, Renmin Avenue, Meilan District, Haikou, 570228, China

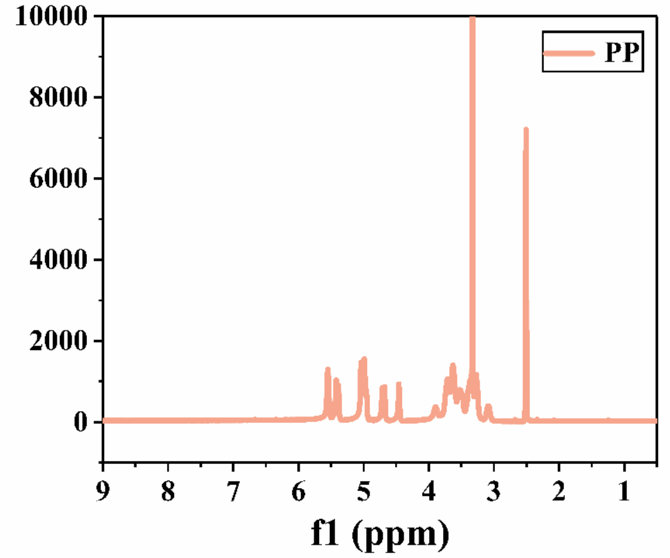

Figure S1. ^{1}H NMR spectrum of pullulan polysaccharides.

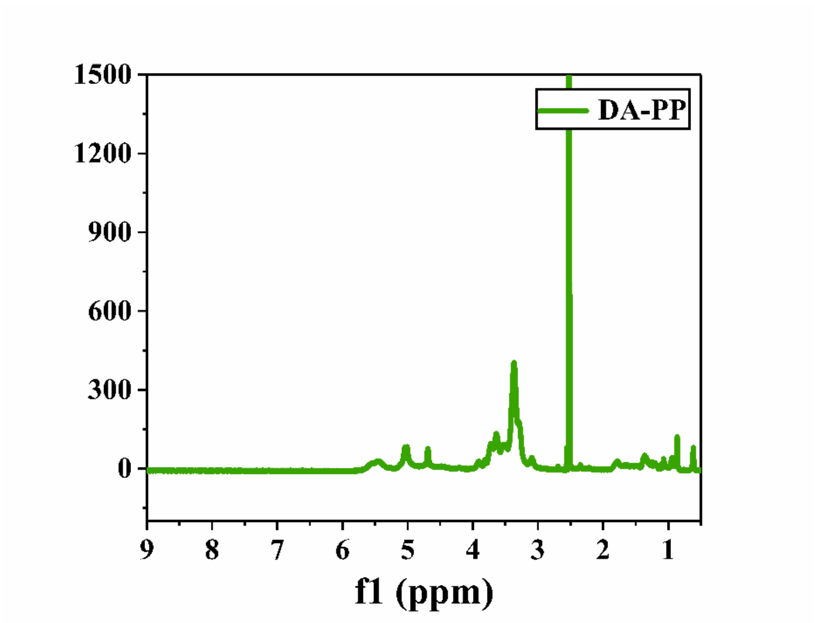

Figure S2. ^{1}H NMR spectrum of polymer DA-PP.

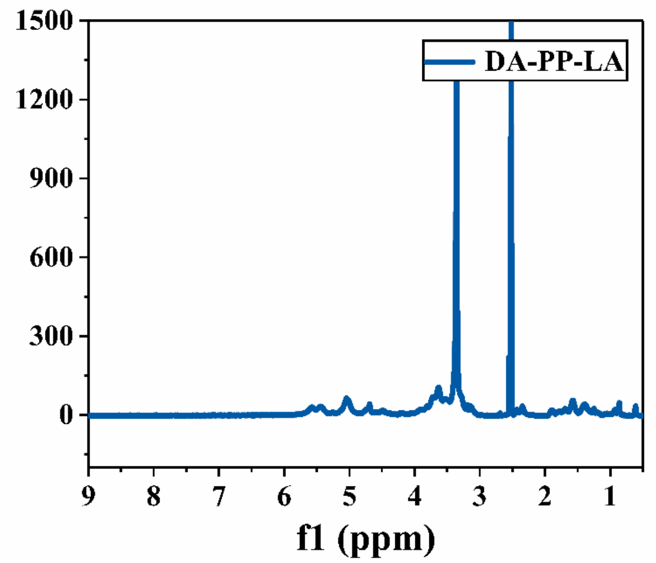

Figure S3. ^{1}H NMR spectrum of polymer DA-PP-LA.

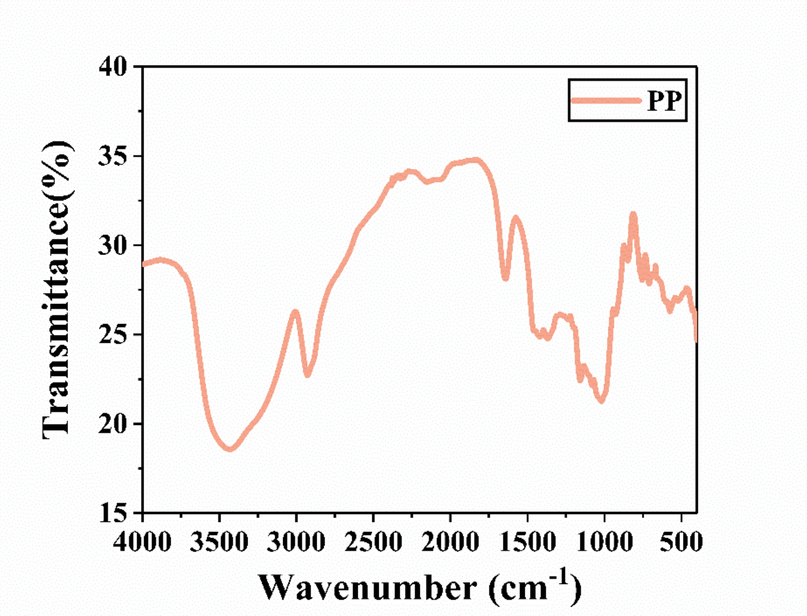

Figure S4. Infrared spectrum of pullulan polysaccharides.

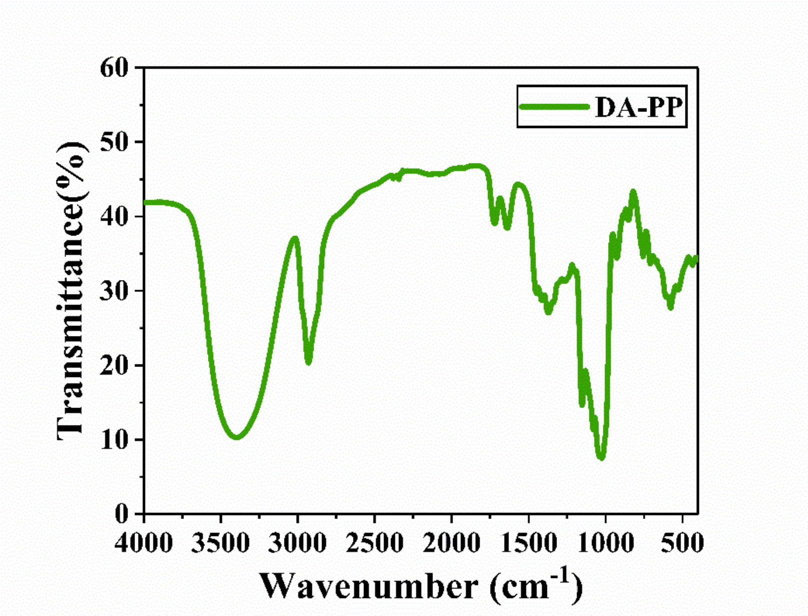

Figure S5. Infrared spectrum of polymer DA-PP.

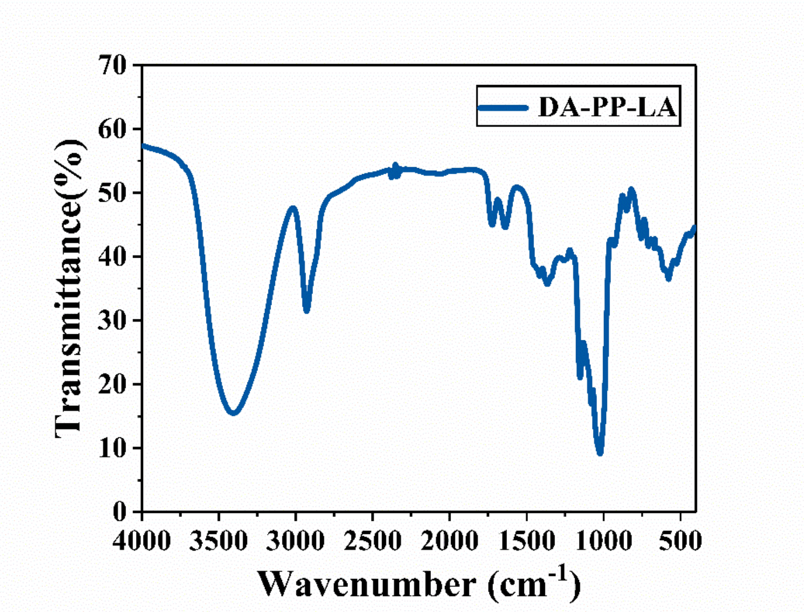

Figure S6. Infrared spectrum of polymer DA-PP-LA.
